# Wolcott-Rallison Syndrome Due to a Novel Mutation (R491X) in EIF2AK3 Gene

**DOI:** 10.4274/Jcrpe.619

**Published:** 2012-06-09

**Authors:** Ercan Mıhçı, Doğa Türkkahraman, Sian Ellard, Sema Akçurin, İffet Bircan

**Affiliations:** 1 Akdeniz University School of Medicine, Department of Pediatrics, Division of Clinical Genetics, Antalya, Turkey; 2 Antalya Education and Research Hospital, Department of Pediatric Endocrinology, Antalya, Turkey; 3 Institute of Biomedical Science, Peninsula Medical School, University of Exeter, UK; 4 Akdeniz University School of Medicine, Department of Pediatric Endocrinology, Antalya, Turkey; +90 242 249 65 17+90 242 227 43 20emihci@akdeniz.edu.tr

**Keywords:** Wolcott-Rallison syndrome, neonatal diabetes, Skeletal dysplasia

## Abstract

Wolcott-Rallison syndrome (WRS) is a rare autosomal recessive disordercharacterized by early-onset diabetes, spondyloepiphyseal dysplasia,tendency to skeletal fractures secondary to osteopenia, and growthretardation. Mutations in the eukaryotic translation initiation factor 2αkinase (EIF2AK3)gene are responsible for this disorder. Here, wedescribe a boy with neonatal diabetes, diagnosed at 2 months of age,who developed severe growth retardation and a skeletal fracture duringthe follow-up period. The patient’s skeletal X-ray revealed findings ofskeletal dysplasia. A clinical diagnosis of WRS was confirmed by theidentification of a novel homozygous nonsense mutation (R491X) in exon9 of the EIF2AK3 gene. The aim of this report is to raise the awarenessfor Wolcott-Rallison syndrome in cases presenting with isolatedneonatal diabetes. This patient demonstrates that the other findings ofthis syndrome might be obscured by a diagnosis of isolated neonataldiabetes.

**Conflict of interest:**None declared.

## INTRODUCTION

Wolcott-Rallison syndrome (WRS) is a rare autosomal recessive disorder characterized by early-onset diabetes mellitus, skeletal dysplasia (especially multiple epiphyseal dysplasia), osteoporosis and growth retardation (1). Other occasional findings of WRS include central hypothyroidism, hepatic dysfunction, renal insufficiency, central nervous system abnormalities, cardiorespiratory defects, and hypothalamic-pituitary dysfunction (2,3,4,5,6,7). To date, less than 100 cases have been described (8). Mutations in the eukaryotic translation initiation factor 2α kinase (EIF2AK3) gene are responsible for this disorder (9). The EIF2AK3 gene regulates protein synthesis by phosphorylating the α-subunit of the eukaryotic initiation factor-2 (eIF2) in the endoplasmic reticulum and is specifically required in the insulin-secreting β-cells during fetal life and early neonatal period (10). Here, we report a novel nonsense mutation (R491X) in the EIF2AK3 gene in a patient with WRS.

## CASE REPORT

The male proband is an infant of Turkish ancestry who was born at 39 weeks gestation by caesarean section with a birth weight of 2300g (<3^rd^ percentile, small for gestational age). His parents were second-degree cousins. Family history was unremarkable. At 2 months of age, the patient had been diagnosed to have neonatal diabetes. Diabetes-associated auto-antibodies were negative. He was discharged with a regimen of NPH and regular insulin injections twice a day.At age 5.5 years, the patient was still on insulin therapy(0.9 U/kg/day). His HbA1c level was 7.7%. Mental-motor development was normal. His height percentile was below 3rd percentile (height SD score: -2.6) and his growth velocity was low (3.6 cm/year). Thyroid function tests were normal, and anti-gliadin IgA was negative for celiac disease. Bone age was retarded by 1.5 years. He had proportionate short stature, and had no sign of skeletal abnormalities.At age 7 years, the patient was admitted to our hospital with a right-sided femur fracture following a simple trauma.

**Figure 1:**
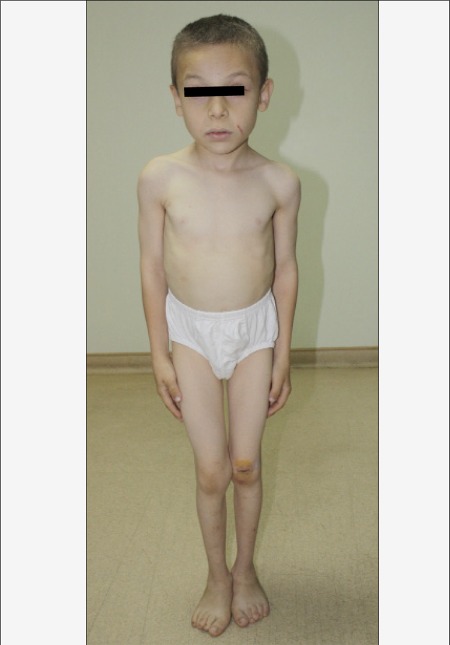
Photograph of the patient demonstrating posture

**Figure 2:**
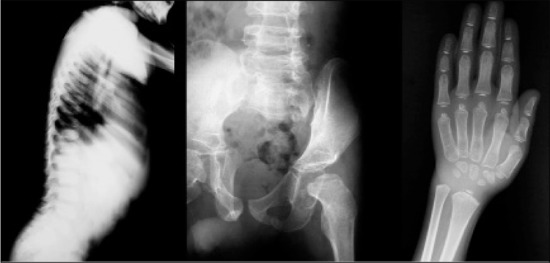
X-ray films demonstrating beaking of both thoracic and lumbarvertebrae, spina bifida at L5 vertebrae, generalized epiphysealmetaphysealdysplasia and osteopenia of the skeletal system

X-ray films showed findings consistent with skeletal dysplasia. Additionally, he had a protuberant abdomen, lumbar lordosis and extension limitation in all joints (Figure 1). Height was severely retarded (102.6 cm, height SD score: -3.6). Growth velocity was 2 cm per year despite his better metabolic control. X-rays revealed beaking of both thoracic and lumbar vertebrae, spina bifida at L5 vertebrae and generalized epiphyseal-metaphyseal dysplasia (Figure 2). Dual-energy X-ray absorptiometry (DEXA) showed osteopenia according to height-age (L2-4 Z-score: -1.6), and abdominal ultrasonography revealed hypoplasia of the pancreas (severely atrophic). Cerebellar atrophy was detected on cranial magnetic resonance imaging.

**Figure 3:**
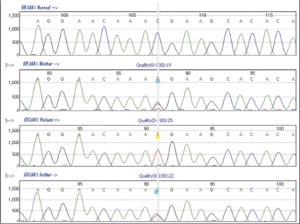
Identification of the mutation in the EIF2AK3 gene. Sequencesshow normal DNA, heterozygous parents, and the findings in the patient

These findings led us to re-evaluate the diagnosis of this patient. WRS was considered as the most plausible diagnosis because of coexistence of neonatal diabetes and skeletal abnormalities. At this time, the patient was found to have elevated liver enzyme values. However, these values decreased and returned to normal within a week and were linked to an episode of viral infection. Genomic DNA was extracted from peripheral leukocytes using standard procedures. The coding exons and the intron-exon boundaries of the EIF2AK3 gene were PCR-amplified; primers and conditions are available upon request. Sequence-specific primers for each amplicon were tagged with 5' M13 tails to allow sequencing to be performed with a "universal" M13 primer. Single-strand sequencing was performed using standard methods on an ABI 3730 (Applied Biosystems, Warrington, UK). Sequences were compared to the published template (accession number AF110146.1) using Mutation Surveyor v3.24 (SoftGenetics, PA, USA). Any changes in the sequence were checked against published polymorphisms and mutations and for conservation across species. Sequence variants were tested for their presence in family members whenever a DNA sample was available.Sequencing analysis has shown that the patient is homozygous for a novel nonsense mutation, R491X (c.1471C>T; p.Arg491X), in exon 9 of the EIF2AK3 gene. Both his mother and father were heterozygous carriers for this mutation (Figure 3). 

## DISCUSSION

Permanent neonatal diabetes mellitus (PNDM) is a very rare clinical condition defined as diabetes diagnosed within the first 6 months of life that does not remit. The estimated incidence of PNDM is 1 in 260 000 and it is most commonly caused by the mutations in the KCNJ11, ABCC8 and INS genes (11,12,13,14).

The mutations in the gene encoding EIF2AK3 are responsible for WRS. Our patient was found to be homozygous for a novel nonsense mutation (R491X) in exon 9 of the EIF2AK3 gene. His mother and father, who were second cousins, were confirmed as heterozygous carriers. A recent study has shown that WRS is the most common cause of PNDM in consanguineous pedigrees (8). In addition to testing patients with a definite clinical diagnosis, these authors recommend that EIF2AK3 mutations should be sought in patients with isolated neonatal diabetes diagnosed after 3 weeks of age from known consanguineous families, isolated populations, or countries where inbreeding is common.

In the literature, the major skeletal abnormalities of WRS are listed as osteoporosis, osteopenia, thoracolumbar kyphosis and bowing of the femora (6). In our patient, osteopenia, beaked thoracic and lumbar vertebrae, a spina bifida at L5 vertebrae and generalized epiphyseal-metaphyseal dysplasia were all present.

Various central nervous system anomalies including cerebellar cortical dysplasia, cerebral atrophy and pachygyria have been described in patients with WRS (6,15,16). Our patient was also found to have cerebellar hypoplasia, which is one of the very rare central nervous system abnormalities of WRS. Severe pancreatic hypoplasia with prominent islets of Langerhans, an important finding of WRS, was present in our patient as well. Notably, in our patient, even though we regulated his blood glucose levels with intensive insulin therapy, and excluded other possible pathologies, growth velocity remained very low. Low growth velocity in such patients might be an initial finding for suspecting WRS in patients presenting with subtle skeletal abnormalities.

In conclusion, the findings in this patient indicate that WRS can present initially as neonatal diabetes mellitus, obscuring the other findings of this syndrome. On the other hand, especially in consanguineous families, even when there is no evidence of skeletal abnormalities, the diagnosis of WRS should be kept in mind in infants/children with growth retardation.
